# Eplerenone Prevents Cardiac Fibrosis by Inhibiting Angiogenesis in Unilateral Urinary Obstruction Rats

**DOI:** 10.1155/2022/1283729

**Published:** 2022-09-17

**Authors:** Yi Chang, Ying Ben, Hui Li, Yunzhao Xiong, Gege Chen, Juan Hao, Xuelian Ma, Xiaomeng Gao, Panpan Qiang, Tatsuo Shimosawa, Xiangting Wang, Fan Yang, Qingyou Xu

**Affiliations:** ^1^Institute of Integrative Medicine, College of Integrative Medicine, Hebei University of Chinese Medicine, Shijiazhuang 050200, China; ^2^Hebei Key Laboratory of Integrative Medicine on Liver-Kidney Patterns, Hebei University of Chinese Medicine, Shijiazhuang 050091, China; ^3^Graduate School, Hebei University of Chinese Medicine, Shijiazhuang 050091, China; ^4^Department of Clinical Laboratory, School of Medicine, International University of Health and Welfare, Narita, Chiba 108-8329, Japan

## Abstract

**Introduction:**

Cardiovascular disease constitutes the leading cause of mortality in patients with chronic kidney disease (CKD), which is termed cardiorenal syndrome type 4 (CRS-4). Here, we report the development of pathological cardiac remodeling and fibrosis in unilateral urinary obstruction (UUO) rats.

**Methods:**

Hematoxylin and eosin (H&E) staining was performed to observe the pathology of myocardial tissue. The degree of myocardial tissue fibrosis was observed by Masson and Sirius red staining. Immunohistochemical staining was applied to detect the expression of CD34 and CD105 in myocardial tissue, and immunofluorescent staining was performed to examine the expression of CD34, collagen I/collagen III, and alpha smooth muscle actin (*α*-SMA). The expression of the signal pathway-related proteins vascular endothelial growth factor A (VEGFA), vascular endothelial growth factor receptor 2 (VEGFR2), nuclear factor *κ*B (NF-*κ*B), and interleukin (IL)-1*β* was tested by western blotting. Reverse transcription-polymerase chain reaction (RT-PCR) was used to detect the mRNA levels of serum and glucocorticoid-inducible kinase (SGK)-1, NF-*κ*B, and interleukin-1*β* (IL-1*β*).

**Results:**

The results showed the development of pathological cardiac remodeling and cardiac dysfunction in UUO rats. Moreover, there was more angiogenesis and endothelial-mesenchymal transition (End-MT) in the UUO group, and these effects were inhibited by eplerenone.

**Conclusions:**

The results indicated that this cardiac fibrosis was associated with angiogenesis and that End-MT was related to aldosterone and mineralocorticoid receptor (MR) activation. Moreover, in association with the MR/IL-1*β*/VEGFA signaling pathway, early treatment with the MR antagonist eplerenone in rats with UUO-induced CKD may significantly attenuate MR activation and cardiac fibrosis.

## 1. Introduction

Chronic kidney disease (CKD), characterized as renal dysfunction, is recognized as a major public health problem with high morbidity and mortality from noncommunicable disease. In 2017, 697.5 million cases of all-stage CKD were recorded, for a global prevalence of 9.1% [1]. Cardiovascular disease (CVD) is the most common complication of CKD and the leading cause of more than 50% of deaths in patients with CKD [2]. CKD that leads to cardiac abnormalities is referred to as cardiorenal syndrome type 4 (CRS-4). Various manifestations of chronic renal impairment-induced myocardial injury in CRS-4 include left ventricular hypertrophy (LVH), diastolic dysfunction, and decreased cardiac function, effects that increase the risk of death among CKD patients [3]. Cardiovascular events are the leading cause of death in patients with CKD, increasing the risk by 10 to 20 times, yet CRS4-related cardiac pathology remains poorly understood [4].

Cardiac hypertrophy and fibrosis are frequently observed in cardiomyopathy associated with CKD and end-stage renal disease (ESRD). During the development of hypertrophy, interstitial cells, such as capillary endothelial cells and cardiac fibroblasts, also undergo dynamic phenotypic changes to support the contractile function of the myocardium [5]. Cardiomyocyte hypertrophy, while cardiomyocytes secrete a variety of vascular growth factors, such as vascular endothelial growth factor (VEGF), which can stimulate angiogenesis to meet the myocardial blood supply demand. Therefore, there is a close relationship and a dynamic balance between cardiomyocytes and new blood vessels. However, continuous angiogenesis can lead to maladaptive ventricular remodeling, leading to myocardial fibrosis and heart failure.

In recent years, there have been an increasing number of studies on the molecular mechanism between angiogenesis and cardiomyocytes, but the role of angiogenesis in the occurrence and development of cardiac fibrosis is still not very clear. It is currently believed that chronic inflammation and excessive activation of the renin-angiotensin-aldosterone system (RAAS) play a key role in this process. Moreover, aldosterone and activation of the mineralocorticoid receptor (MR) also cause cardiac inflammation and fibrosis, vascular fibrosis and remodeling, tubulointerstitial fibrosis, and glomerular injury. Therefore, in this study, we focused on the role of angiogenesis in CRS-4 and the mechanisms that mediate the associated cardiac pathology.

## 2. Materials and Methods

### 2.1. Animal Model, Grouping, and Administration

Male Wistar rats (Hebei Medical University Animal Center, SCXK 2018–004, *N* = 30) weighing 170 ± 10 g were used in this study. The rats were maintained on standard rat chow and tap water at 22°C under a 12 h light/12 h dark cycle. The animal experiment was approved by the Ethics Committee for Animal Experimentation of the Hebei University of Chinese Medicine (Hebei, China), approval number DWLL2021063.

The rats were randomly assigned to three groups: the sham group, UUO group, and UUO with eplerenone (EPL) treatment group. There were 10 animals in each group. For the UUO operation, the rats were anesthetized with 1.5% isoflurane by continuous inhalation. The left kidney was exposed through a left flank incision, and then the left ureter was ligated with 5-0 silk at two sites between the bladder and renal pelvis. After surgery, eplerenone (Pfizer, United States) was given to UUO rats in the EPL-treated group via a diet at a dose of 1.25 g/kg diet (100 mg/kg/day) for 6 months, and the other groups of rats were fed regular chow. Echocardiograms were performed via a Vevo 2100 system (VisualSonics, Canada) 6 months after UUO, prior to harvesting tissues. Renal function was evaluated by blood urea nitrogen (BUN) and serum creatinine (Scr), which were measured by screening kits from Beckman Coulter. Hearts were collected, weighed, and processed for histological examination and protein and mRNA tests.

### 2.2. Echocardiography Analysis

All rats were anesthetized with 3% isoflurane and underwent echocardiographic measurements using a Vevo 2100 system (VisualSonics, Canada). The echocardiographic parameters included heat rate, left ventricular anterior wall (LVAW), left ventricular internal dimension (LVID), left ventricle posterior wall thickness (LVPW), ejection fraction (EF), and fractional shortening (FS).

### 2.3. Histological Analysis and Immunohistochemistry Staining

The hearts were harvested and fixed overnight in 4% paraformaldehyde and dehydrated with alcohol for paraffin embedding. Heart sections (4 *μ*m) were stained with H&E, Masson's trichrome, Sirius red, and immunohistochemistry for *α*-smooth muscle actin (*α*-SMA, ABclonal, A17910, 4000000298, China), CD34 (Abcam, ab81289, GR3240236-22, UK), CD105 (Abcam, ab2529, GR3256475-11, UK), and NR3C2 (Proteintech, 00016122, USA). Sections were observed by microscopy (Leica, BX53, Germany). Positive cells and the staining area were quantified in 10 consecutive high-power fields per heart using ImageJ version 1.8.0 and expressed as the number of positive cells/view or percent positive area.

### 2.4. Immunofluorescence Staining

After fixation, the hearts were incubated in 20% sucrose overnight and then embedded in optimal cutting temperature compound (OCT). Cryosections of heart tissue were stored at -80°C until use. (6 *μ*m) Heart sections were rehydrated in phosphate-buffered saline (PBS) for 5 minutes. After washing, the sections were blocked with 10% goat serum for 20 minutes. The sections were incubated with primary antibodies at 4°C overnight and again with secondary antibody at 37°C for 1 hour. The primary antibodies used for immunofluorescence included anti-CD34 (Abcam, UK, ab81289, 1 : 200), anti-CD105 (Abcam, UK, ab2529 1 : 200), anti-*α*-SMA (Abcam, UK, ab202509, GR3283221-3, 1 : 400), anti-collagen I (Abcam, UK, ab270993, GR3394622-8, 1 : 100), and anti-collagen III (Abcam, UK, ab7778, GR3250987-1, 1 : 100). Cell nuclei were stained with 4′,6-diamidino-2-phenylindole (DAPI). All images were obtained using a confocal microscope (Leica, SP8, Germany).

### 2.5. Protein Extraction and Western Blot Analysis

The left ventricle of heart tissue was homogenized in radioimmunoprecipitation assay (RIPA) buffer for protein extraction. A total of 20~50 *μ*g of protein was separated by 10% SDS–PAGE and then transferred to a 0.22 *μ*m polyvinylidene difluoride (PVDF) membrane. The membrane was blocked for 1.5 h at room temperature with 5% milk. Then, the membrane was incubated overnight at 4°C with anti-interleukin-1*β* (IL-1*β*) (NOVUS, 25270, 1 : 1000, China), anti-nuclear factor *κ*B (NF-*κ*B) (Abcam, UK, 1 : 1000, GR200963-20), anti-NR3C2 (Proteintech, USA, 21854-1-AP, 00016122 1 : 1000), anti-vascular endothelial growth factor A (VEGFA) (Abcam, UK, GR3194799-3, 1 : 1000), and anti-vascular endothelial growth factor receptor 2 (VEGFR2) (Abcam, UK, 1 : 500) antibodies and incubated with fluorescein-conjugated secondary antibodies (LI-COR, USA, C80605-11; LI-COR, USA, C80710-11) for 1 hour at room temperature. GAPDH antibody was used for normalization of protein loading. The protein expression was scanned with an Odyssey Infrared Imaging System (LI-COR, Lincoln, NE, USA).

### 2.6. Real-Time Quantitative PCR Analysis

Total RNA in the left ventricle of heart tissues was extracted using the EZNA. Total RNA Kit II (Omega, Bio-Tek, Norcross, GA, USA) according to the manufacturer's protocol, and reverse transcription was performed using the MonScriptTM RT III all-in-one Mix (Monad Biotech, China). The cDNA was used as a template for MonAmpTM ChemoHS qPCR Mix (Monad Biotech, China), and real-time qPCR analysis was performed by the ABI 7500 FAST System (Applied Biosystems, USA). The genes (and the sequences of the primers used) are as follows: SGK-1 (forward 5′-CTTCTGTGGCACGCCTGAGTATC-3′, reverse 5′-AGCCTCTTGGTCCGG TCCTTC-3′); IL-1*β* (forward 5′-ACAGCAGCATCTCGACAAGAGC-3′, reverse 5′-CCACGGGC AAGA-CATAGGTAGC-3′); NF-*κ*B (forward 5′-TTTTCAGCACTGATTATAGCAGGTT-3′, reverse 5′-AAGGTATCGCAGTCCCCACC-3′); and GAPDH (forward 5′-GTCCATGCCATCACTGCCACTC-3′, reverse 5′-CGCCTGCTTCACCACCTTCTTG-3′).

### 2.7. Statistical Analysis

The values are expressed as the means ± standard deviations (SD). Statistical analysis was performed with SPSS 23.0 (IBM, USA) and GraphPad Prism version 8.0 (GraphPad Software, USA). Statistical comparisons were performed with one-way ANOVA followed by Tukey's *post hoc test* for multiple groups, and Student's *t*-test was used for two groups. Tests were performed to determine whether the data were normally distributed. A significance level of *p* < 0.05 was defined as being statistically significant.

## 3. Results

### 3.1. Chronic Kidney Injury and Cardiac Dysfunction Induced by Unilateral Urinary Obstruction (UUO)

**Figure 1:**
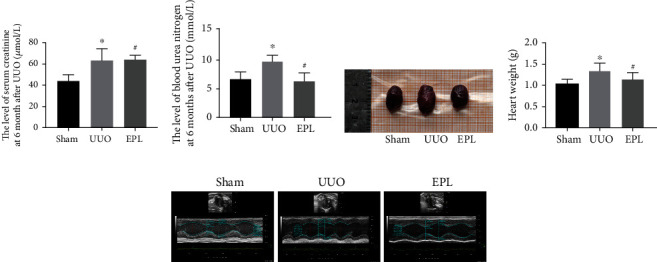
Chronic kidney injury and cardiac dysfunction are induced by UUO. (a) Scr in serum was evaluated for function. (b) BUN in serum was evaluated for renal function. (c) Representative images of hearts harvested from all groups. (d) Cardiac mass as measured by the heart weight from all groups. (e) Echocardiography images from all groups. Each value represents the mean ± SD. *N* = 6. ^∗^*p* < 0.05 vs. sham. ^#^*p* < 0.05 vs. UUO.

UUO is a well-known model of CKD [6]. Six months after left UUO, we observed the effects of chronic kidney injury and cardiac function. Renal function tests for BUN and Scr levels revealed impaired renal function in long-term UUO rats compared with sham rats (shown in Figures 1(a) and 1(b)). Cardiac hypertrophy was observed in rats with UUO-induced CKD, as indicated by images of hearts and increased heart weight (*p* < 0.05, shown in Figures 1(c) and 1(d)). Echocardiography was employed at 6 months after UUO. LVIDs and LVs were increased in rats with UUO-induced CKD, and EF was decreased (*p* < 0.05, shown in Figure 1(e), Table 1). Notably, treatment of UUO rats with EPL protected kidney and cardiac function (shown in Figures 1(a) and 1(b), Table 1). The results showed that UUO rats can serve as models of CKD and develop cardiac dysfunction, and an MR antagonist, EPL, can protect both renal and cardiac function.

### 3.2. UUO Induces Cardiac Fibrosis and Inflammation in Rats

**Figure 2:**
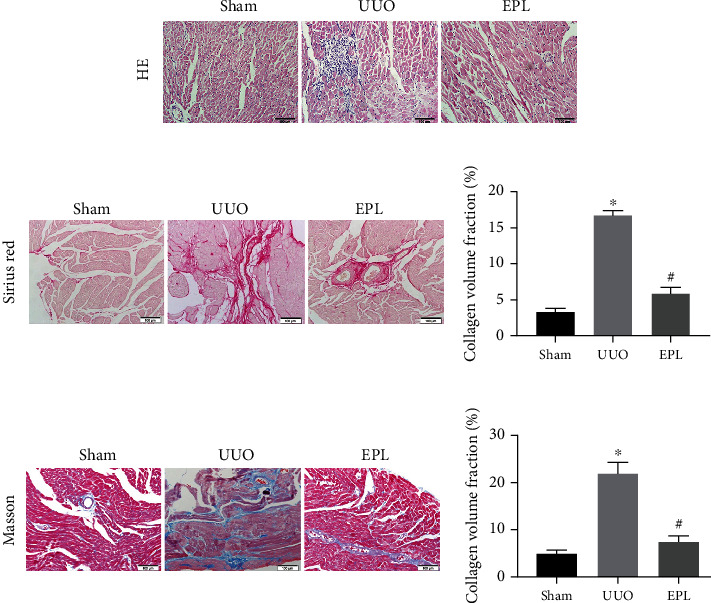
UUO induces cardiac fibrosis and inflammation in rats. (a) Heart sections were stained with HE for morphological changes. (b) Sirius red staining for collagen deposition. (c) Masson's trichrome staining for collagen deposition. The fibrosis area is enlarged. Scale bars, 100 *μ*m. Each value represents the mean ± SD. *N* = 6. ^∗^*p* < 0.05 vs. Sham. ^#^*p* < 0.05 vs. UUO.

H&E staining of the heart showed injured myocardial cells, irregular nuclear morphology, uneven staining, and increased inflammatory cell infiltration in the myocardium of UUO rats (shown in Figure 2(a)). Moreover, Sirius red staining showed significantly more collagen deposition and fibrosis in the myocardial interstitium (*p* < 0.05, shown in Figure 2(b)), and the same results were seen in Masson's trichrome staining (*p* < 0.05, shown in Figure 2(c)). However, the pathological changes and collagen deposition were alleviated in EPL-treated rats (shown in Figure 2). These findings suggest that UUO-induced CKD in rats causes cardiac dysfunction and cardiac fibrosis, which is similar to the pathophysiology of CRS-4. The results imply that this model can be used to investigate the pathophysiology of CRS-4. EPL decreased cardiac fibrosis and reversed dysfunction, which suggests that aldosterone plays a key role in CRS-4.

### 3.3. Angiogenesis and Endothelial-Mesenchymal Transition (End-MT) Promote Cardiac Fibrosis in UUO Rats

**Figure 3:**
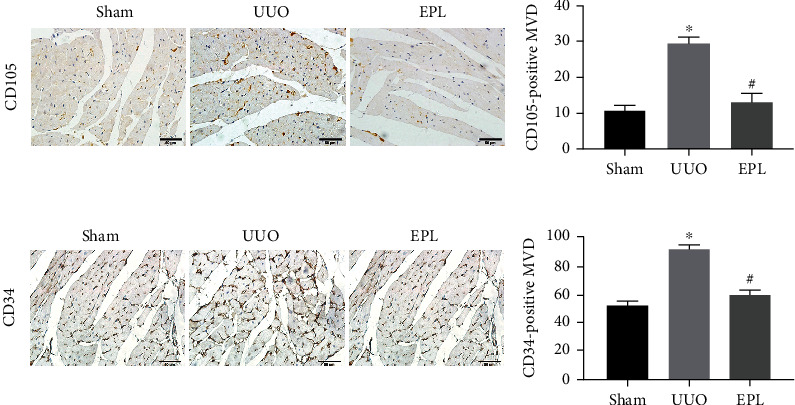
CD105-positive MVD and CD34-positive MVD increased in the hearts of UUO rats. (a) Immunohistochemistry staining using antibodies against CD105 to examine neovascular endothelial cells in cardiac tissues. (b) Immunohistochemistry staining with antibodies against CD34 to examine endothelial cells in cardiac tissues. MVD: microvascular density. Scale bars, 50 *μ*m. Each value represents the mean ± SD. *N* = 6, ^∗^*p* < 0.05 vs. sham.^#^*p* < 0.05 vs. UUO.

We further examined whether angiogenesis is associated with the process of cardiac fibrosis. We therefore compared the number of endothelial cells and neovascular endothelial cells in the heart from the three groups. CD34 is a marker of endothelial cells and is expressed in large amounts in the myocardial tissues of rats. CD105 is a marker of neovascular endothelial cells. Immunohistochemical analysis showed that the numbers of CD105^+^ cells (*p* < 0.05, shown in Figure 3(a)) and CD34^+^ cells (*p* < 0.05, shown in Figure 3(b)) were significantly increased in the myocardial tissue of the UUO group compared with the sham group. This finding suggests that there was more angiogenesis in the UUO group than in the sham group.

**Figure 4:**
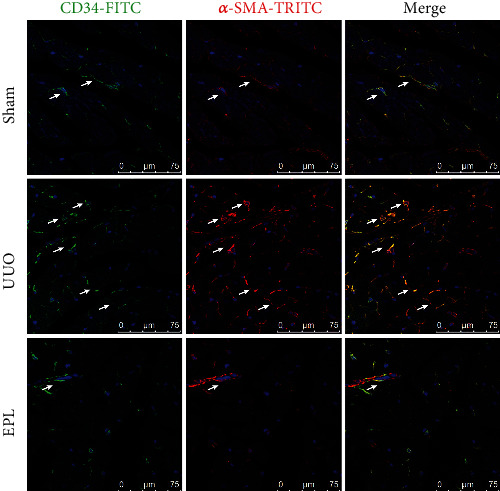
The endothelial-mesenchymal transition (End-MT) was increased in UUO rats. Immunofluorescent multistaining with antibodies against endothelial cell marker CD34 (FITC, green) and myofibroblast marker *α*-SMA (TRITC, red) for identifying End-MT (cells coexpressing the two markers indicate End-MT, and nuclei were stained with DAPI in blue). Scale bars, 75 *μ*m.

**Figure 5:**
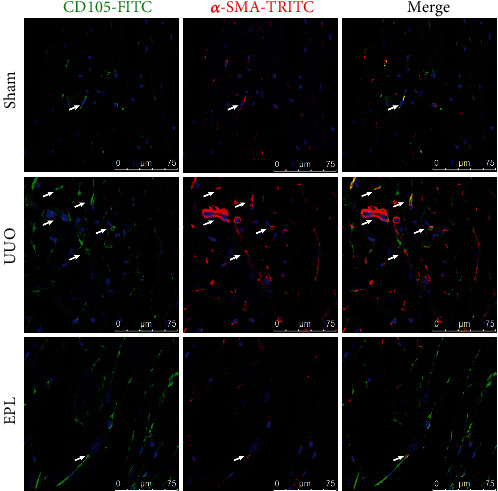
The neovascular endothelial-mesenchymal transition was increased in UUO rats. Immunofluorescent multistaining with antibodies against neovascular endothelial cell marker CD105 (FITC, green) and myofibroblast marker *α*-SMA (TRITC, red) for identifying the neovascular endothelial-mesenchymal transition (cells coexpressing the two markers, and nuclei were stained with DAPI in blue). Scale bars, 75 *μ*m.

**Figure 6:**
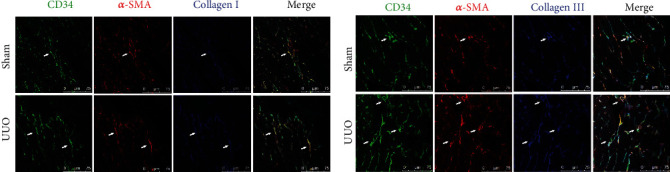
End-MT cells produce collagen type I (COL I) and collagen type III (COL III). (a) Immunofluorescent multistaining identifies cells coexpressing CD34 (FITC, green), *α*-SMA (TRITC, red), and COL I (Alexa Fluor® 405, blue). Scale bars, 75 *μ*m. (b) Immunofluorescent multistaining identifies cells coexpressing CD34 (FITC, green), *α*-SMA (TRITC, red), and COL III (Alexa Fluor® 405, blue). Scale bars, 75 *μ*m.

Cardiac fibrosis is the main pathological change in CRS-4. We speculated that End-MT plays a key role in this process. There are a large number of neovascular endothelial cells. To clarify the role of End-MT in the formation of myocardial fibrosis, we further evaluated the coexpression of CD34/CD105 and *α*-SMA, a marker of myofibroblasts, by immunofluorescence. The results showed more CD34^+^/*α*-SMA^+^ positive cells and CD105^+^/*α*-SMA^+^ positive cells in the UUO group than in the sham group (shown in Figures 4 and 5). End-MT cells play an important role in cardiac fibrosis by secreting collagen, and collagen I (Col I) and collagen III (Col III) are the most abundant collagen components in the myocardial interstitium. Three-color confocal microscopy analysis identified Col I and Col III secretion around CD34^+^/*α*-SMA^+^cells (shown in Figure 6). The inhibitory effect of EPL on angiogenesis and End-MT may indicate that the activation of MR is related to cardiac fibrosis.

### 3.4. Eplerenone Inhibits Activated MR-Induced Angiogenesis by Regulating the VEGFRA/VEGFR2 Pathway

**Figure 7:**
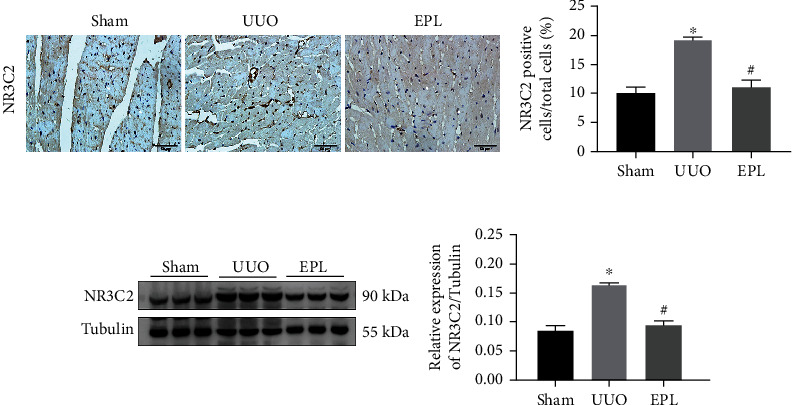
MRs were activated in UUO injury rats. (a) Immunohistochemistry staining identifies NR3C2 expression in the nucleus. Scale bars, 50 *μ*m. Each value represents the mean ± SD. *N* = 6. ^∗^*p* < 0.05 vs. Sham. ^#^*p* < 0.05 vs. UUO. (b) The expression of NR3C2 protein in cardiac tissue of UUO rats was detected by western blot. Each value represents the mean ± SD. *N* = 3. ^∗^*p* < 0.05 vs. Sham. ^#^*p* < 0.05 vs. UUO.

Aldosterone works through activation with MR, and we confirmed MR activation by examining the expression of NR3C2 by immunohistochemistry and western blotting. NR3C2 is located mainly in the cytoplasm and interacts with heat shock protein 90 (HSP90); then, when MR is activated, NR3C2 is translocated to the nucleus to mediate gene transcription. We found that more NR3C2 was transferred to the nucleus (*p* < 0.05, shown in Figure 7(a)) and that there was more expression of NR3C2 (*p* < 0.05, shown in Figure 7(b)) in the UUO group than in the sham group; these effects were inhibited by eplerenone (shown in Figure 7).

**Figure 8:**
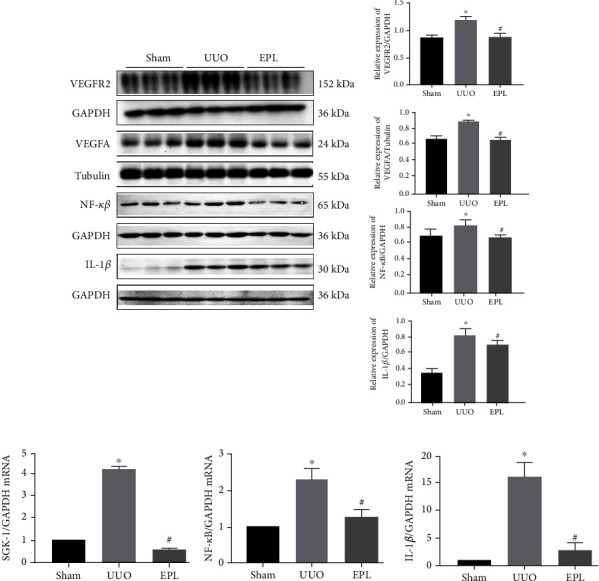
Aldosterone stimulates angiogenesis, and End-MT is activated by the MR/IL-1*β*/VEGFA signaling pathway. (a) The protein expression of NF-*κ*B, IL-1*β*, VEGFA, and VEGFR2 in cardiac tissue of UUO rats was detected by western blot. Each value represents the mean ± SD. *N* = 3. ^∗^*p* < 0.05 vs. sham. ^#^*p* < 0.05 vs. UUO. (b) The SGK-1, NF-*κ*B, and IL-1*β* mRNA levels were detected via real-time PCR. Each value represents the mean ± SD. *N* = 3. ^∗^*p* < 0.05 vs. Sham. ^#^*p* < 0.05 vs. UUO.

To further investigate the signaling pathway of angiogenesis and End-MT related to MR activation, we used western blot and real-time qPCR to test the signaling pathway protein and mRNA levels. We found that the protein expression of NF-*κ*B, IL-1*β*, VEGFA, and VEGFR2 was upregulated in the injured hearts of UUO rats and downregulated by eplerenone treatment (*p* < 0.05, shown in Figure 8(a)). Moreover, the same results were found for the mRNA levels of SGK-1, NF-*κ*B, and IL-1*β* (*p* < 0.05, shown in Figure 8(b)).

## 4. Discussion

CRS-4 defines cardiovascular involvement in patients with CKD. It has been acknowledged that patients with advanced kidney disease are at high risk of cardiovascular disease morbidity and mortality [7]. Furthermore, it is currently being advocated that patients with earlier stages of CKD also suffer a high rate of cardiovascular events, and CKD is now considered an independent CVD risk factor [8]. Although an increasing number of studies have investigated the pathogenesis of CRS-4 in recent years, the underlying pathophysiology is complex and has not yet been clearly described [9].

Our study utilized the UUO model for 6 months of long-term observation, which induces chronic kidney and cardiac injury. UUO is a valuable model to elucidate both the pathogenesis and the mechanisms responsible for progressive renal fibrosis. Our data revealed that the UUO rats caused certain adverse effects on the structure and function of the myocardium, especially the decreased LVEF. The CRS-4 model was successfully replicated. CKD caused a certain degree of fibrosis of the myocardium, and the contraction function of the myocardium also decreased to a certain extent. Although it has been studied in a short-term UUO model for CRS-4, myocardial fibrosis, and cardiac hypertrophy were observed, and cardiac dysfunction did not develop [10, 11]. Similarly, the 5/6 nephrectomy (5/6Nx) surgical model of CKD has been shown to induce cardiac remodeling and vascular changes at the determined endpoint in another study [12]. The model of CRS generally involves the establishment of heart injury, kidney injury, or combined heart and kidney injury. Most of the CKD models used in the CRS-4 study use nephrectomy or bilateral ischemia–reperfusion injury to cause severe renal insufficiency [13, 14], which has a high mortality rate. In the current study, we used a long-term UUO model in which CKD is developed and can simulate the long-term onset of CRS-4 with a lower mortality rate, which is essential in the clinic as acute and chronic urinary obstruction due to benign prostatic hypertrophy, renal calculi, or other urinary retention [10].

Angiogenesis is a complex process of budding and the formation of new blood vessels from preexisting microvessels via migration, proliferation, and survival. In ischemic coronary artery diseases, such as acute myocardial infarction (AMI) and ischemic cardiomyopathy, angiogenesis increases the blood supply to tissues [15]. However, studies on angiogenesis and CRS-4 have rarely been reported, especially long-term UUO-induced cardiac fibrosis. Interestingly, we also detected angiogenesis during the observation of cardiac injury in UUO rats. In rats with UUO-induced CKD, the endothelial cell marker CD34 and neovascular endothelial cell marker CD105 were abundant. The results showed high levels of CD34 and CD105 expression and an increase in vascular endothelial cells in the left ventricle. Cardiac hypertrophy and fibrosis are the main pathological changes in CRS-4. Pathological hypertrophy is also associated with cardiac structural remodeling and myocardial fibrosis, and sustained pathological hypertrophy leads to congestive heart failure [16]. During the development of hypertrophy, capillary endothelial cells, and cardiac fibroblasts dynamically undergo phenotypic changes to support the contractile function of the myocardium, especially angiogenesis with capillary microvasculature, and myocytes increased in proportion to the hypertrophy [17, 18]. However, disproportional cardiac myocyte growth and angiogenesis may lead to myocardial ischemia and promote adverse cardiac fibrosis.

A key regulator of angiogenesis is vascular endothelial growth factor (VEGF), which stimulates angiogenesis by acting on VEGF receptor 2 (VEGFR2) on endothelial cells [19]. In the heart, VEGF is produced by cardiac myocytes in response to hypoxia-induced factor 1 (HIF-1*α*) [5], a key mediator during periods of ischemic insult, and is also triggered by hemodynamic forces and inflammation. Activation of the RAAS has a significant effect on cardiorenal connectors. In particular, aldosterone is a key factor in inflammation and adverse cardiovascular remodeling by acting on the MR [20]. Furthermore, an increasing number of studies have shown that aldosterone participates in endothelial dysfunction, vascular fibrosis, and inflammation in the vasculature [21, 22]. Inflammation is a common driver of pathological cardiac remodeling after cardiac injury [23]. During cardiac injury and remodeling, the immune system is activated, and immune cells, such as T lymphocytes, B lymphocytes, and macrophages, can promote fibroblast activation by secreting inflammatory cytokines, leading to abnormal collagen metabolism and thus causing myocardial fibrosis [24]. There is also a strong relationship between inflammation and fibrosis in other organs [25]. Similarly, aldosterone has been suggested to play an important role in angiogenesis [26]. Eplerenone, a selective aldosterone blocker, is clinically used for the treatment of CRS-4 and heart failure. Although numerous studies related to the effects of aldosterone and eplerenone on cardiovascular disease have been performed, the relationship between aldosterone and angiogenesis in the development of CRS-4 is not yet clear and has conflicting results. Michel et al. showed for the first time that aldosterone increases neovascularization in the setting of ischemia through activation of Ang II signaling [27]. Similarly, one study showed that aldosterone plays a pivotal role in hepatocellular carcinoma development through VEGF-mediated tumor angiogenesis and that eplerenone attenuates angiogenesis in mice [28]. However, some studies have revealed that aldosterone inhibits tube formation of endothelial cells (ECs) and suppresses angiogenesis [29, 30]. In our study, EPL successfully reversed angiogenesis and cardiac dysfunction. This suggests that MRs might function upstream of angiogenesis. Aldosterone activates MRs to induce inflammatory changes in the heart, and it is possible that inflammation activates VEGF and VEGFR2 transcription. In the current study, we showed that VEGF and VEGFR2 colocalize with other MR target genes, such as SGK1, and inflammatory markers, such as NF-*κ*B and IL-1*β*. We did not directly examine the impact of aldosterone and MRs on vascular endothelial cells in our research. Thus, further studies are required to verify this hypothesis and understand the mechanism of MRs in endothelial cells.

However, whether angiogenesis plays a good or bad role is unclear in CRS-4, which is a long and chronic process of cardiac fibrosis. Myofibroblasts secrete large amounts of extracellular matrix (ECM) proteins, such as collagens and fibronectin, which contribute to heart remodeling and myocardial fibrosis [31]. Although cardiac fibroblasts are considered essential modulators of the ECM network, several other cell types, including immune cells, vascular endothelial cells, and cardiomyocytes, have been implicated in ECM remodeling either directly or indirectly [32]. The fibroblast-myofibroblast transition and cell proliferation increase Col I and Col III secretion. Otherwise, End-MT, which is characterized by losing the endothelial phenotype and obtaining myofibroblastic properties, also contributes to the deposition of ECM [33, 34]. In our research, we found more CD34^+^/*α*-SMA^+^ positive cells and CD105^+^/*α*-SMA^+^ positive cells in the UUO group than in the sham group, as well as Col I and Col III deposition. Therefore, we speculate that End-MT also occurs in neovascular endothelial cells to participate in the pathophysiological process of myocardial fibrosis. However, we observed angiogenesis only 6 months after UUO injury, and the role of angiogenesis in myocardial fibrosis could be a gradual change over time.

## 5. Conclusion

In conclusion, our study demonstrates the development of pathological cardiac remodeling and cardiac dysfunction in UUO rats. Angiogenesis and End-MT may play important roles in cardiac hypertrophy and fibrosis, both of which are regulated by activated MR-induced upregulation of the VEGFA/VEGFR2 signaling pathway. The MR antagonist eplerenone can protect cardiomyocytes and decrease End-MT, which highlights the importance of MR antagonists for preclinical CRS-4.

## Figures and Tables

**Table 1:** Echocardiography of sham rats, UUO injury rats, and eplerenone-treated rats.

Quantitative parameters	Sham	UUO	EPL
HR(bmp)	368.21 ± 17.31	330.72 ± 32.42^∗^	354.99 ± 40.21
LVEDd(mm)	6.92 ± 0.66	7.70 ± 0.42	6.76 ± 0.63^#^
LVEDs(mm)	3.72 ± 0.52	4.60 ± 0.39^∗^	3.28 ± 0.50^#^
LVPWd(mm)	1.59 ± 0.11	1.55 ± 0.10	1.70 ± 0.21
LVPWs(mm)	2.72 ± 0.17	2.56 ± 0.18^∗^	2.98 ± 0.20^#^
LVM(mg)	594.44 ± 116.64	700.95 ± 83.70	654.13 ± 36.26
LVd(*μ*L)	251.49 ± 54.79	313.22 ± 37.89	238.02 ± 48.88^#^
LVs(*μ*L)	60.46 ± 20.07	98.19 ± 19.10^∗^	44.97 ± 15.27^#^
LVEF(%)	77.32 ± 2.84	69.12 ± 4.23^∗^	80.52 ± 3.61^#^
FS(%)	46.36 ± 3.13	40.26 ± 3.52^∗^	50.52 ± 3.81^#^

HR: heart rate; LVEDd: left ventricular end-diastolic diameter; LVESd: left ventricular end-systolic diameter; LVAWd: left ventricular anterior wall dimension at end-diastole; LVPWd: left ventricular posterior wall thickness at end-diastole; LVM: left ventricular mass; LVd: left ventricular diastolic volume; LVs: left ventricular systolic volume; LVEF: left ventricular ejection fraction; FS: fractional shortening. ^∗^*p* < 0.05 vs. sham. *N* = 6. ^#^*p* < 0.05 vs. UUO.

## Data Availability

The original contributions presented in the study are included in the article/Supplementary Material, and further inquiries can be directed to the corresponding authors.
